# Microbial Functional Responses Explain Alpine Soil Carbon Fluxes under Future Climate Scenarios

**DOI:** 10.1128/mBio.00761-20

**Published:** 2021-02-23

**Authors:** Qi Qi, Yue Haowei, Zhenhua Zhang, Joy D. Van Nostrand, Linwei Wu, Xue Guo, Jiajie Feng, Mengmeng Wang, Sihang Yang, Jianshu Zhao, Qun Gao, Qiuting Zhang, Mengxin Zhao, Changyi Xie, Zhiyuan Ma, Jin-Sheng He, Haiyan Chu, Yi Huang, Jizhong Zhou, Yunfeng Yang

**Affiliations:** a State Key Joint Laboratory of Environment Simulation and Pollution Control, School of Environment, Tsinghua University, Beijing, China; b Ecological Environment Bureau, Shenzhen, China; c Key Laboratory of Adaptation and Evolution of Plateau Biota, Northwest Institute of Plateau Biology, Chinese Academy of Sciences, Xining, China; d Institute for Environmental Genomics, Department of Microbiology and Plant Biology, and School of Civil Engineering and Environmental Sciences, University of Oklahoma, Norman, Oklahoma, USA; e School of Life Sciences, South China Normal University, Guangzhou, China; f Institute of Public Safety Research, Department of Engineering Physics, Beijing Key Laboratory of City Integrated Emergency Response Science, Tsinghua University, Beijing, China; g Department of Ecology, College of Urban and Environmental Sciences, and Key Laboratory for Earth Surface Processes of the Ministry of Education, Peking University, Beijing, China; h State Key Laboratory of Soil and Sustainable Agriculture, Institute of Soil Science, Chinese Academy of Sciences, Nanjing, China; i State Key Joint Laboratory of Environmental Simulation and Pollution Control, College of Environmental Science and Engineering, Peking University, Beijing, China; j Earth and Environmental Sciences Division, Lawrence Berkeley National Laboratory, Berkeley, California, USA; University of Massachusetts Amherst; University of Massachusetts Amherst

**Keywords:** carbon flux, warming, altered precipitation, alpine grassland, plant-microbe-soil interaction

## Abstract

Soil microorganisms are sensitive to temperature in cold ecosystems, but it remains unclear how microbial responses are modulated by other important climate drivers, such as precipitation changes. Here, we examine the effects of six *in situ* warming and/or precipitation treatments in alpine grasslands on microbial communities, plants, and soil carbon fluxes. These treatments differentially affected soil carbon fluxes, gross primary production, and microbial communities. Variations of soil CO_2_ and CH_4_ fluxes across all sites significantly (*r *>* *0.70, *P* < 0.050) correlated with relevant microbial functional abundances but not bacterial or fungal abundances. Given tight linkages between microbial functional traits and ecosystem functionality, we conclude that future soil carbon fluxes in alpine grasslands can be predicted by microbial carbon-degrading capacities.

## INTRODUCTION

Soil is the largest repository of organic carbon in the terrestrial biosphere and represents an important source of CO_2_ and CH_4_, releasing more than 70 Pg carbon to the atmosphere annually (1, 2). Soil carbon fluxes are strongly influenced by warming, as soil CO_2_ flux increased at an average yearly rate of 0.1 Pg carbon from 1989 to 2008 (3), and global CH_4_ flux increased at an average yearly rate of 27 Tg carbon from 2008 to 2017 (4). However, warming decreases soil CO_2_ flux in dryland ecosystems due to microbial thermal acclimation and reduced enzymatic activities (5). Inconsistent responses of soil carbon fluxes to warming can partially be attributed to concurrent changes in precipitation (1, 2, 6, 7), which regulate the metabolic capacity of fine roots and microbial activities (8). Consequently, the temperature dependence of carbon fluxes is modulated by soil moisture (9), indicating the important role of the interaction between temperature and moisture. This complexity has generated considerable uncertainty in predicting carbon fluxes under future climate conditions (3, 8, 9).

Both plants and soil-dwelling microbes dually regulate the flux of carbon in and out of the soil, so their responses to warming and precipitation impose significant changes to soil carbon fluxes. Drying generally decreases plant photosynthesis (7), while the effect of warming depends on leaf diffusive conductance (10, 11). These climate changes also substantially affect microbial communities (12). They can affect microbial enzyme activities (13) and microbial community composition through changes in plant functional groups and soil environments (14, 15), which in turn affect soil carbon decomposition rates (16).

Short-term warming affects microbial functional composition but not the taxonomic composition of microbial communities in Arctic tundra, suggesting that microbial functional traits were more sensitive to warming (17). In the Arctic, microbial functional composition was significantly correlated with soil respiration or CH_4_ flux (18). However, it is unclear whether the patterns between microbial functional composition and carbon fluxes observed in high latitude ecosystems are still true in high altitude ecosystems.

To examine microbial and plant roles in mediating carbon fluxes, we carried out a field experiment in the alpine grassland of the Tibetan Plateau, a highly sensitive ecosystem (19) in which the warming rate (0.2°C per decade) dwarfs the global warming rate by a factor of two (20). Precipitation in Tibet is jointly controlled by summer monsoons and the North Atlantic Oscillation, which is projected to increase precipitation in Southern and Northern areas of the Plateau and decrease precipitation in the central Plateau (21). Therefore, we set up a study site in July 2011, which contains two levels of warming (control, warming +2°C) crossed with three levels of precipitation (control, wetting +50%, and drying −50%). By measuring soil, plant, and microbial variables, we sought to test two hypotheses: (i) whether microbial functional capacities associated with carbon cycling are differentially affected by warming and precipitation, and (ii) whether variations in soil carbon fluxes and net ecosystem exchange (NEE) are tightly linked to microbial functional traits rather than microbial taxonomic composition. We adopted a functional gene microarray, GeoChip, to detect a wide variety of functional genes (22). We analyzed bacterial communities by amplicon sequencing of bacterial 16S rRNA genes and determined the operational taxonomic units (OTU) by clustering sequences at 97% similarity. We analyzed fungal communities by amplicon sequencing of fungal internal transcribed spacer (ITS) regions and determined the OTU by clustering sequences at 97% similarity.

## RESULTS

### Effects of climate changes on plant and soil variables.

Warming increased gross primary production (GPP; +20.39%, *P* < 0.050) and ecosystem respiration (ER; +26.03%, *P* < 0.050; see Table S1 in the supplemental material). Wetting increased GPP (+23.74%, *P* < 0.050) and decreased net ecosystem exchange (NEE; −29.36%, *P* < 0.050). As a result, a larger soil carbon sink was observed, consistent with the finding that Tibet had served as a carbon sink since 2000 (23). In contrast, drying caused very little change to plant and soil variables (see Table S1 in the supplemental material). Combined warming and wetting stimulated soil CO_2_ flux (+19.22%, *P* < 0.050) and decreased NEE (−56.94%, *P* < 0.050), while combined warming and drying increased root/shoot ratio (+61.50%, *P* < 0.050) and decreased soil water content (SWC, −31.61%, *P* < 0.050), aboveground net primary production (ANPP; −37.69%, *P* < 0.050), and plant richness (−19.67%, *P* < 0.050). Combined warming and drying also dramatically decreased ecosystem multifunctionality (EMF; −289.47%, *P* < 0.050), defined as the ability of ecosystems to maintain multiple services and functions such as carbon storage and productivity (24). Plants not only are the source of carbon for microbes but also contribute to soil CO_2_ flux and NEE directly by root respiration. Therefore, we measured belowground net primary production (BNPP) and found that it remained unchanged by any treatment (see Table S1). No interactive effects of warming and precipitation were detected for all of 15 soil variables except for pH (see Table S2).

10.1128/mBio.00761-20.1 TABLE S1 Environmental variables. Download Table S1, DOCX file, 0.02 MB. Copyright © 2021 Qi et al. 2021 Qi et al. https://creativecommons.org/licenses/by/4.0/ This content is distributed under the terms of the Creative Commons Attribution 4.0 International license.

10.1128/mBio.00761-20.2 TABLE S2 Treatment effects on environmental variables and microbial communities. Download Table S2, DOCX file, 0.03 MB. Copyright © 2021 Qi et al. 2021 Qi et al. https://creativecommons.org/licenses/by/4.0/ This content is distributed under the terms of the Creative Commons Attribution 4.0 International license.

### Effects of climate changes on microbial communities.

Although most treatments did not affect microbial biomass, combined warming and drying decreased soil microbial biomass carbon (MBC; −27.37%, *P* < 0.050) and microbial biomass nitrogen (MBN; −30.08%, *P* < 0.050; see Table S1) considerably, suggesting that soil microbial communities were under stress. Combined warming and drying also decreased the MBC-normalized abundances (i.e., the relative abundances of functional genes based on GeoChip intensity multiplied by MBC) of a majority of the microbial functional genes associated with carbon degradation and CH_4_ cycling (see Fig. S1). These genes included *ftr* encoding ferredoxin-thioredoxin reductase, *mcrA* encoding *α-*subunit of methyl-coenzyme M reductase, and *mmox* encoding a methane monooxygenase. Archaeal sequences related to both hydrogenotrophic and acetoclastic methanogens were detected, suggesting that CH_4_ could originate from both CO_2_ reduction and acetate fermentation.

10.1128/mBio.00761-20.8 FIG S1 MBC-normalized abundances of microbial functional genes associated with carbon degradation (a) and methane cycling (b). Functional genes associated with starch degradation (*amyA*, *amyX*, *apu*, *cda*, *glucoamylase*, *isopullulanase*, *nplT*, and *pulA*), hemicellulose degradation (*ara*, *mannanase*, *xylA*, and *xylanase*), cellulose degradation (*axe*, *cellobiase*, *GH7 cellulase*, *endoglucanase*, and *exoglucanase*), chitin degradation (*acetylglucosaminidase*, *chitin deacetylase*, and *chitinase*), lignin degradation (*glx*, *ligninase*, *mnp*, and *phenol oxidase*), methanogenesis (*fwdB*, *ftr*, *hmd*, *mch*, *mtrH*, *mer*, *acs*, *mtaB*, *mtmB*, *mtbC*, *mcrA*, *MT2*, and *hdrB*). and methane oxidation (*mmoX* and *pmoA*) are shown. Different alphabets on the bars were calculated by *post hoc* Tukey’s honestly significant difference test with a linear mixed-effects model (*P* < 0.050). Download FIG S1, DOCX file, 0.6 MB. Copyright © 2021 Qi et al. 2021 Qi et al. https://creativecommons.org/licenses/by/4.0/ This content is distributed under the terms of the Creative Commons Attribution 4.0 International license.

The taxonomic and functional *α-*diversities remained unchanged by any treatment (see Fig. S2a). The main effect of precipitation alteration (*F *=* *1.24 to 2.67, *P* < 0.050), rather than warming (*F *=* *0.71 to 1.27, *P > *0.050), was significant on overall functional and taxonomic composition (PerMANOVA; see Table S2). There were few interactive effects of warming and precipitation on microbial communities. The functional dissimilarity of within-group samples, calculated as the mean Bray-Curtis weighted dissimilarities in functional composition between pairs of replicates in a group, was increased by warming (see Fig. S2b), consistent with a divergent succession of soil microbial communities under warming (25).

10.1128/mBio.00761-20.9 FIG S2 (a and b) α-diversity (a) and Bray-Curtis dissimilarities of within-group samples (b) of soil microbial communities under different treatments. Different alphabets on the bars associated with *α*-diversity were calculated by *post hoc* Tukey’s honestly significant difference test with a linear mixed-effects model (*P* < 0.050). Different alphabets on the bars associated with dissimilarities of within-group samples were calculated by Kruskal-Wallis H test (*P* < 0.050). Download FIG S2, DOCX file, 0.3 MB. Copyright © 2021 Qi et al. 2021 Qi et al. https://creativecommons.org/licenses/by/4.0/ This content is distributed under the terms of the Creative Commons Attribution 4.0 International license.

### Strong linkages between soil carbon fluxes and microbial functional abundances.

**FIG 1:**
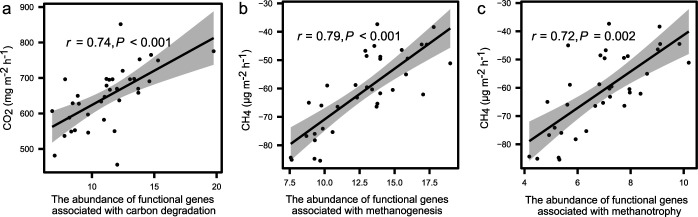
Correlations between carbon fluxes and the MBC-normalized abundances of microbial functional genes. (a) Orrelation between soil CO_2_ flux and the MBC-normalized abundances of functional genes associated with carbon degradation. (b and c) Correlation between soil CH_4_ flux and the MBC-normalized abundance of functional genes associated with methanogenesis (b) and methane oxidation (c).

The functional composition of microbial communities, rather than the taxonomic composition, was significantly associated with soil CO_2_ flux (*r *=* *0.48, *P* < 0.001; partial Mantel tests in Table 1), CH_4_ flux (*r *=* *0.54, *P < *0.001) and other ecosystem functions such as ER (*r *=* *0.54, *P < *0.001), GPP (*r *=* *0.28, *P = *0.006), and NEE (*r *=* *0.40, *P = *0.002). Moreover, soil CO_2_ flux increased with abundances of functional genes associated with carbon degradation (*r *=* *0.74, *P < *0.001; Fig. 1a), while soil CH_4_ flux increased with abundances of functional genes associated with CH_4_ cycling (*r *>* *0.72, *P < *0.002; Fig. 1b and c). Consistently, soil CO_2_ flux increased with abundances of individual functional genes associated with carbon degradation, such as *xylA* encoding xylose isomerases (*r *=* *0.69, *P < *0.001; see Fig. S3a). The soil CH_4_ flux increased with abundances of individual functional genes associated with CH_4_ cycling, such as *mcrA* (*r *=* *0.77, *P < *0.001), encoding α*-*subunit of methyl coenzyme M reductase and *pmoA* encoding *α-*subunit of particulate methane monooxygenase (*r *=* *0.70, *P < *0.001; see Fig. S3b). Since functional abundances were normalized by MBC, we repeated the correlational analyses using relative abundances of functional genes without normalization. Significant correlations between abundances and carbon fluxes were still observed (see Table S3), excluding the possibility that microbial biomass determined the correlations. In contrast, there were few significant correlations between carbon fluxes and MBC-normalized abundances (i.e., relative abundance of 16S rRNA gene/ITS multiplied by MBC) at a fine taxonomic (i.e., the genus) or a coarse taxonomic (i.e., the phylum) resolution (see Table S4).

**TABLE 1:** Partial Mantel tests between environmental variables and microbial communities

Environmental variable^*a*^	*R* value (*P*)^*b*^		
	Microbial functional composition	Bacterial community composition	Fungal community composition
Clay (%)	0.37 (0.002)**	0.02 (0.341)	−0.07 (0.783)
Silt (%)	0.28 (0.032)*	−0.06 (0.668)	0.03 (0.389)
Sandy (%)	0.33 (<0.001)***	0.07 (0.189)	−0.05 (0.716)
pH	0.08 (0.198)	−0.04 (0.621)	−0.12 (0.875)
SWC (g kg^−1^)	0.19 (0.047)*	−0.03 (0.590)	−0.08 (0.805)
Temp (°C)	−0.09 (0.844)	0.04 (0.302)	0.13 (0.055)
TC (g kg^−1^)	0.01 (0.369)	0.01 (0.415)	0.03 (0.359)
TN (g kg^−1^)	0.01 (0.385)	0.04 (0.323)	0.07 (0.267)
TP (g kg^−1^)	0.19 (0.043)*	−0.03 (0.578)	−0.08 (0.824)
SOC (g kg^−1^)	−0.04 (0.671)	0.11 (0.145)	0.03 (0.347)
NH_4_^+^ (mg kg^−1^)	−0.02 (0.538)	−0.18 (0.981)	−0.10 (0.854)
NO_3_^–^ (mg kg^−1^)	0.02 (0.380)	−0.02 (0.578)	−0.02 (0.578)
SAP (mg kg^−1^)	0.23 (0.007)**	0.24 (0.011)*	0.02 (0.400)
DOC (mg kg^−1^)	0.11 (0.139)	0.07 (0.251)	0.07 (0.251)
DON (mg kg^−1^)	−0.06 (0.702)	−0.14 (0.956)	−0.09 (0.826)
CO_2_ (mg m^−2^ h^−1^)	0.48 (<0.001)***	0.15 (0.084)	0.02 (0.427)
CH_4_ (μg m^−2^ h^−1^)	0.54 (<0.001)***	−0.19 (0.974)	−0.06 (0.672)
N_2_O (μg m^−2^ h^−1^)	0.38 (0.017)*	−0.22 (0.994)	−0.06 (0.695)
BNPP (g m^−2^)	0.17 (0.074)	−0.21 (0.994)	−0.18 (0.981)
NEE (μmol CO_2_ m^−2^ s^−1^)	0.40 (0.002)**	0.16 (0.073)	0.03 (0.350)
ER (μmol CO_2_ m^−2^ s^−1^)	0.54 (<0.001)***	−0.19 (0.983)	−0.06 (0.662)
GPP (μmol CO_2_ m^−2^ s^−1^)	0.28 (0.006)**	0.18 (0.067)	0.15 (0.087)
Grass ANPP (g m^−2^)	0.06 (0.223)	−0.08 (0.722)	−0.08 (0.722)
Sedge ANPP (g m^−2^)	0.09 (0.156)	0.09 (0.161)	0.07 (0.275)
Forb ANPP (g m^−2^)	0.13 (0.073)	0.03 (0.369)	0.07 (0.215)
Other ANPP (g m^−2^)	−0.09 (0.758)	0.03 (0.358)	0.02 (0.404)
ANPP (g m^−2^)	0.12 (0.128)	0.01 (0.396)	−0.08 (0.743)
Total plant biomass	0.79 (0.079)	−0.00 (0.477)	0.02 (0.396)
Root/shoot ratio	−0.03 (0.594)	−0.04 (0.647)	0.01 (0.414)
Grass richness	0.37 (<0.001)***	0.10 (0.141)	−0.06 (0.788)
Sedge richness	−0.07 (0.792)	0.23 (0.008)**	0.11 (0.060)
Forb richness	0.13 (0.096)	0.18 (0.054)	0.06 (0.280)
Other plant richness	−0.01 (0.474)	0.10 (0.189)	0.13 (0.136)
Plant richness	0.37 (<0.001)***	0.13 (0.062)	0.04 (0.324)
Plant diversity	0.05 (0.264)	0.09 (0.169)	0.04 (0.346)
EMF	0.27 (<0.001)***	0.08 (0.206)	−0.11 (0.872)

a Abbreviations: clay, soil clay content, silt, soil silt content, sandy, soil sandy content; SWC, soil water content; temp, soil temperature; TC, soil total carbon; TN, soil total nitrogen; TP, soil total phosphorus; SOC, soil organic carbon; SAP, soil available phosphorus; DOC, dissolved organic carbon; DON, dissolved organic nitrogen; CO_2_, soil CO_2_ flux; CH_4_, soil CH_4_ flux; N_2_O, soil N_2_O flux; BNPP, belowground net primary production; NEE, net ecosystem exchange; ER, ecosystem respiration; GPP, gross primary productivity; grass ANPP, the aboveground net primary production of grass; sedge ANPP, the aboveground net primary production of sedge; Forb ANPP, the aboveground net primary production of forb; other ANPP, the aboveground net primary production of other functional groups of plant; ANPP, the aboveground net primary production of total plant community; total plant biomass, the sum of aboveground and belowground net primary production; root/shoot ratio, the ratio of BNPP to ANPP; grass richness, species number of grass; sedge richness, species number of sedge; Forb richness, species number of forb; other plant richness, species number of other functional groups of plants; plant richness, the species numbers of plant community; plant diversity, the α*-*diversity of plant communities based on calculation of Shannon index; EMF, ecosystem multifunctionality.

b *, *P* < 0.050; **, *P* < 0.010; ***, *P* < 0.001.

10.1128/mBio.00761-20.3 TABLE S3 Correlations between carbon fluxes and relative abundances of microbial functional genes. Download Table S3, DOCX file, 0.02 MB. Copyright © 2021 Qi et al. 2021 Qi et al. https://creativecommons.org/licenses/by/4.0/ This content is distributed under the terms of the Creative Commons Attribution 4.0 International license.

10.1128/mBio.00761-20.4 TABLE S4 Correlations between carbon fluxes and the MBC-normalized abundances of major bacterial and fungal taxa. Download Table S4, DOCX file, 0.02 MB. Copyright © 2021 Qi et al. 2021 Qi et al. https://creativecommons.org/licenses/by/4.0/ This content is distributed under the terms of the Creative Commons Attribution 4.0 International license.

10.1128/mBio.00761-20.10 FIG S3 Correlations between soil carbon fluxes and the MBC-normalized abundances of microbial functional genes. (a) Correlations between soil CO_2_ flux and the MBC-normalized abundances of microbial functional genes associated with starch degradation (*amyA*, *amyX*, *apu*, *cda*, *glucoamylase*, *isopullulanase*, *nplT*, and *pulA*), hemicellulose degradation (*ara*, *mannanase*, *xylA*, and *xylanase*), cellulose degradation (*axe*, *cellobiase*, *GH7 cellulase*, *endoglucanase*, and *exoglucanase*), chitin degradation (*acetylglucosaminidase*, *chitin deacetylase*, and *chitinase*), lignin degradation (*glx*, *ligninase*, *mnp*, and *phenol oxidase*). (b) Correlations between soil CH_4_ flux and the MBC-normalized abundances of microbial functional genes associated with methanogenesis (*fwdB*, *ftr*, *hmd*, *mch*, *mtrH*, *mer*, *acs*, *mtaB*, *mtmB*, *mtbC*, *mcrA*, *MT2*, and *hdrB*) and methanotrophy (*mmoX* and *pmoA*). Download FIG S3, DOCX file, 0.6 MB. Copyright © 2021 Qi et al. 2021 Qi et al. https://creativecommons.org/licenses/by/4.0/ This content is distributed under the terms of the Creative Commons Attribution 4.0 International license.

### Strong linkages between soil carbon fluxes and microbial functional genes.

We analyzed individual contribution of microbial and plant communities to carbon fluxes. Microbial functional genes, soil, and selected plant variables of ANPP, BNPP, and plant Shannon diversity were significantly linked with soil CO_2_ flux (partial Mantel tests; Table 2). In contrast, only microbial functional genes and soil variables, but not selected plant variables, were significantly linked with soil CH_4_ flux (*P < *0.050). We also performed multiple regression on distance matrices (MRM; see Table S5), which showed significant linkages between microbial functional genes and carbon fluxes (*P < *0.050). Furthermore, we selected important categories of microbial functional genes and found that microbial functional groups associated with recalcitrant carbon degradation were significantly linked with soil CO_2_ flux and NEE (partial Mantel tests; Table 3). In addition, the explanatory powers of functional genes associated with carbon degradation derived from bacteria or fungi for soil CO_2_ flux and NEE are stronger than those of bacterial or fungal communities (Table 2).

**TABLE 2:** Influence of microbial communities, plant variables, and soil variables on carbon fluxes by partial Mantel tests^*a*^

Variable^*b*^	CO_2_		Variable	CH_4_		Variable	NEE	
	*R*	*P*		*r*	*P*		*r*	*P*
Carbon_degradation	0.35	0.003**	Methane	0.18	0.007**	Carbon_degradation	0.29	0.015*
Bacterial community	0.09	0.187	Bacterial community	−0.04	0.674	Bacterial community	0.12	0.141
Fungal community	0.01	0.501	Fungal community	−0.08	0.837	Fungal community	−0.01	0.538
Plant	0.36	0.002**	Plant	0.13	0.059	Plant	0.21	0.029*
Soil	0.36	<0.001***	Soil	0.48	<0.001***	Soil	0.24	0.003**
Carbon_degradation_B	0.34	0.006**	Methane_B	0.20	0.005**	Carbon_degradation_B	0.28	0.013*
Carbon_degradation_A	0.47	0.001**				Carbon_degradation_A	0.40	0.002**
Carbon_degradation_F	0.48	0.001**				Carbon_degradation_F	0.44	0.001**

a Abbreviations: CO_2_, soil CO_2_ flux; CH_4_, soil CH_4_ flux; NEE, net ecosystem exchange. Significance is indicated by asterisks (*, *P* < 0.050; **, *P* < 0.010; ***, *P* < 0.001).

b Carbon_degradation, microbial functional genes associated with carbon degradation; methane, microbial functional genes associated with methane cycling. Plant variables include aboveground and belowground net primary production and plant Shannon diversity. Soil variables include soil clay content, silt content, sandy content, pH, soil water content, soil temperature, soil total carbon, total nitrogen, total phosphorus, soil organic carbon, ammonia, nitrate, available phosphorus, dissolved organic carbon, and dissolved organic nitrogen. Carbon_degradation_B, microbial functional genes associated with carbon degradation derived from bacteria; Carbon_degradation_A, microbial functional genes associated with carbon degradation derived from archaea; Carbon_degradation_F, microbial functional genes associated with carbon degradation derived from fungi; Methane_B, microbial functional genes associated with methane cycling derived from bacteria.

**TABLE 3:** Influence of microbial functional genes, plant variables, and soil variables on carbon fluxes by partial Mantel tests^*a*^

Variable^*b*^	CO_2_		Variable	CH_4_		Variable	NEE	
	*r*	*P*		*r*	*P*		*r*	*P*
Starch	−0.05	0.658	Methanogenesis	0.10	0.036*	Starch	−0.11	0.846
Hemicellulose	0.10	0.161	Methanotrophy	0.01	0.391	Hemicellulose	0.14	0.161
Cellulose	0.09	0.183	Plant	0.20	0.006**	Cellulose	0.02	0.408
Chitin	0.20	0.013*	Soil	0.47	<0.001***	Chitin	0.20	0.034*
Lignin	0.18	0.033*				Lignin	0.31	0.002**
Plant	0.32	0.003**				Plant	0.17	0.058
Soil	0.35	0.001***				Soil	0.23	0.009**

a Abbreviations: CO_2_, soil CO_2_ flux; CH_4_, soil CH_4_ flux; NEE, net ecosystem exchange. Significance is indicated by asterisks (*, *P* < 0.050; **, *P* < 0.010; ***, *P* < 0.001).

b Starch represents functional genes associated with starch degradation; hemicellulose represents functional genes associated with hemicellulose degradation; cellulose represents functional genes associated with cellulose degradation; chitin represents functional genes associated with chitin degradation; lignin represents functional genes associated with lignin degradation. Plant variables include aboveground and belowground net primary production and plant Shannon diversity. Soil variables include soil clay content, silt content, sandy content, pH, soil water content, soil temperature, soil total carbon, total nitrogen, total phosphorus, soil organic carbon, ammonia, nitrate, available phosphorus, dissolved organic carbon, and dissolved organic nitrogen.

10.1128/mBio.00761-20.5 TABLE S5 Linkages between soil carbon fluxes and microbial functional genes, plant variables, and soil variables based on multiple regression on distance matrices. Download Table S5, DOCX file, 0.02 MB. Copyright © 2021 Qi et al. 2021 Qi et al. https://creativecommons.org/licenses/by/4.0/ This content is distributed under the terms of the Creative Commons Attribution 4.0 International license.

We also generated linear mixed-effects models to verify the linkages with carbon fluxes (see Table S6). The effects of microbial functional gene abundances were significant and larger than those of the plant and soil variables used, based on standardized coefficients in models. The regression models were able to predict more than 60% of the variations in carbon fluxes across all sites.

10.1128/mBio.00761-20.6 TABLE S6 *R*^2^ values and standardized coefficients of predictors based on linear mixed-effects models. Download Table S6, DOCX file, 0.02 MB. Copyright © 2021 Qi et al. 2021 Qi et al. https://creativecommons.org/licenses/by/4.0/ This content is distributed under the terms of the Creative Commons Attribution 4.0 International license.

**FIG 2:**
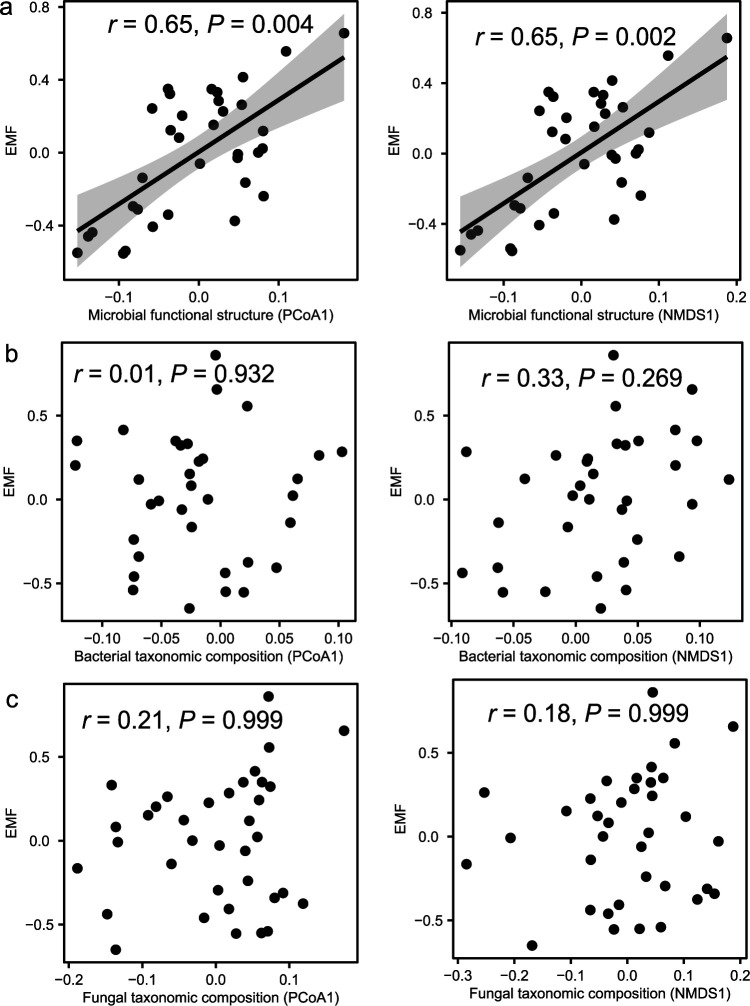
Correlations between EMF and microbial communities. Microbial communities are shown as microbial functional compositions derived from the first axis of principal-coordinate analysis (PcoA1) (a) and derived from the first axis of nonmetric multidimensional scaling (NMDS1) (b) in a bacterial community composition derived from PCoA1 and NMDS1 and derived from a fungal community composition derived from PCoA1 and NMDS1 (c).

We also tested whether there was a linkage between soil microbial communities and EMF. To this end, we conducted principal-coordinate analysis (PCoA) and nonmetric multidimensional scaling (NMDS) for microbial communities. We found that EMF was correlated with the first axis of PCoA/NMDS of microbial functional composition (*r *=* *0.65, *P < *0.004; Fig. 2a) but not with that of bacterial or fungal community composition (*r *<* *0.33, *P* > 0.050; Fig. 2b and c). These results could be verified by partial Mantel tests (Table 1). Procrustes analyses showed that microbial functional composition was not correlated with bacterial taxonomic composition (sum of squares = 0.997, *r *=* *0.05, *P = *0.885) or fungal taxonomic composition (sum of squares = 0.986, *r *=* *0.11, *P = *0.528; see Table S7).

10.1128/mBio.00761-20.7 TABLE S7 Procrustes analyses between microbial functional and taxonomic compositions. Download Table S7, DOCX file, 0.01 MB. Copyright © 2021 Qi et al. 2021 Qi et al. https://creativecommons.org/licenses/by/4.0/ This content is distributed under the terms of the Creative Commons Attribution 4.0 International license.

## DISCUSSION

How microbes and plants contribute to soil carbon fluxes is a fundamental and controversial question related to the fate of soil organic matter (1, 2). Even less is known about climate controls on microbes and plants, consequently affecting soil carbon fluxes. Although warming significantly increased both GPP and abundances of microbial functional genes associated with carbon degradation in tundra ecosystems (17), only GPP was stimulated by warming in our study (see Fig. S1 and Table S1). One possible explanation is that tundra has much higher soil moisture than our sites (17), which could stimulate extracellular enzyme activity of soil microbes to degrade available carbon (26). Warming increased thaw depth and soil moisture in tundra (17) but decreased SWC in the Tibetan plateau (see Table S1). Due to the higher elevation, warming increased soil water evaporation, leading to severe water stress (27).

Whether precipitation has a favorable/detrimental effect on plants and microbes depends on the soil water content, oxygen availability, and soluble substrate supply (7, 27). Generally, ANPP increases with precipitation unless anoxic conditions caused by wetting decrease the rates of nutrients cycling (28). In this study, wetting significantly increased GPP (see Table S1), suggesting that precipitation was a limiting factor for plant growth. In contrast, plants could tolerate drying via enhanced water use efficiency (29), leading to little changes in GPP and ANPP. The soil microbial community composition was also resistant to drying (see Fig. S1), which could be explained by two non-mutually exclusive mechanisms. Microbes can acclimate physiologically to environmental changes by reducing internal solute potential, forming impermeable cell walls, and producing extracellular polymeric substances to survive harsh conditions or go dormant (30, 31). Also, there is the legacy effect of soil moisture, e.g., microbial communities are affected by the previous condition of soil moisture (32). However, the combined warming and drying treatment begot a more severe drought, which significantly decreased ANPP (see Table S1). The total plant biomass was not decreased, owing to an increased plant root/shoot ratio (see Table S1) that offset drought stress and improved drought tolerance (33).

Combined warming and wetting increased soil CO_2_ flux (see Table S1). Consistent with this, combined warming and wetting stimulated the activities of an extracellular enzyme associated with organic matter mineralization (34). In addition, wetting accelerated loss of *p*-coumaric acid and cell wall-bound ferulic (35), which protects carbohydrates by cross-linking with lignin. Consequently, combined warming and wetting stimulated litter decomposition. Since warming causes water limitation for plant and soil microbes (7, 31), combined warming and drying caused stress for the alpine ecosystem, as evidenced by the substantial decrease of ANPP, microbial biomass, and the abundance of microbial functional genes associated with carbon cycling (see Fig. S1 and Table S1).

A meta-analysis study showed that soil CO_2_ flux was positively correlated with ligninase activity, rather than cellulase activity (36). Similarly, we found that soil CO_2_ flux was tightly linked with the microbial functional composition associated with recalcitrant carbon degradation, rather than labile carbon degradation (Table 3). There were significant positive linkages between soil CH_4_ flux and the abundances of microbial functional genes associated with methanogenesis and methanotrophy (Fig. 1b and c), two intertwining processes that jointly contributed to CH_4_ flux (37). Consistent with previous studies showing linkages between soil CH_4_ flux and methanogens (38), the effect of methanogenic community composition on CH_4_ flux was also significant (Table 3). In contrast, we did not observe a significant effect of the methanotrophic community composition on CH_4_ flux, since the methanotrophic community was less affected by warming and precipitation than the methanogenic community (39). Therefore, methanogenic archaea could play a major role in affecting soil CH_4_ flux in the alpine ecosystem. Consistent with our results, more than 80% of soil CH_4_ fluxes could be attributed to methanogenesis regardless of aerobic or anaerobic conditions (40). Our results showed that methanogen abundance is valuable to infer the underlying mechanisms of methane emissions. In addition, a mixed CH_4_ origin suggests the availability of several methanogenic substrates in the alpine grassland, probably derived from the recycling of old carbon (for example, acetate) and H_2_ gas.

Linking microbial communities to ecosystem functioning is a key challenge in microbial ecology (41). Our study demonstrated that EMF was significantly correlated with the microbial functional composition rather than the taxonomic composition (Fig. 2 and Table 1). In accordance, microbial functional composition was decoupled from microbial taxonomy (see Table S7). Functional redundancy among microbial taxa, horizontal gene transfer, convergent evolution, or adaptive loss of microbial function could be responsible for the decoupling (42). Rapid evolution and gene loss caused by horizontal gene transfer can obscure a trait’s phylogenetic signal. Convergent evolution also decreases phylogenetic signal since taxonomically distinct species evolved a similar phenotypic solution due to similar genetic changes or identical mutations (43). In addition, one microbe usually has thousands of functional genes, so functional genes have a higher dimensionality and resolution than that of the 16S rRNA gene (44). The relatively high degree of conservation and low degree of resolution in the 16S rRNA gene make it easy to detect variations in functional genes. In a global soil study, abiotic soil characteristics well explained the variations of microbial gene abundances associated with nitrogen cycling but barely explained the taxonomic composition. These results imply that viewing microbial communities through a taxonomic lens could impair ecological generality (45). For example, most fungi are rather plastic, displaying substantial variation in physiology and morphology that can obscure different life strategies among taxa (46). Given that microbial functional traits can be linked to ecosystem functions (45), analyzing microbial functional capabilities will provide valuable insights into ecosystem functions. Soil CO_2_ fluxes under cold climates are projected to increase with climate warming (47), representing a positive climate-carbon cycle feedback. In alpine grasslands, we showed that changes in precipitation were indispensable for modulating warming-induced microbial functional capacity in carbon degradation. Our study revealed complicated patterns of plant and microbial responses to climate changes, highlighting the importance of taking microbial functional traits into accounts when predicting soil carbon flux changes. By establishing linkages between functional gene abundances and soil carbon fluxes and EMF, we provide novel insights into how microbial functional traits could serve as potential proxies of ecosystem functions.

## MATERIALS AND METHODS

### Field site description and soil sample collection.

Our field site is located at the Haibei Alpine Grassland Ecosystem Research Station in the northeastern Tibet Plateau, China (37°30′N, 101°12′E, 3,200 m above sea level). Soil pH at a depth of 0 to 10 cm is 6.4. The annual mean air temperature at Haibei is −1.5°C. The annual mean precipitation is 501.3 mm. More than 80% of the precipitation occurs from May to September, during which vegetation reaches the maximal aboveground biomass in late July and early August.

Thirty-six experimental plots (six treatments × six replicates) were set up in July 2011, with a completely randomized block design, i.e., random distribution of six treatments, control (C), warming (W), drying (DP), wetting (IP), combined warming and drying (WDP), and combined warming and wetting (WIP) plots. The plots are of 2.2 m in length × 1.8 m in width and separated from other plots by a 4.0-m buffer belt. To stimulate warming of +2°C, two infrared heaters (220 V, 1,200 W) were installed at the height of 1.6 m aboveground in the plots of three warming treatments (W, WIP, and WDP). Dummy heaters were installed for other plots (C, IP, and DP). Four transparent resin polycarbonate channels, which cover 50% of the plot area, are used to intercept precipitation in drying plots (DP and WDP). During rainfall, intercepted precipitation flows into white polyethylene plastic rainwater collection vessels and is then added to wetting plots (IP and WIP), resulting in a 50% precipitation decrease in DP and WDP plots and a 50% precipitation increase in IP and WIP plots. C, IP, and WIP plots also have four transparent channels to mimic the shade effect in the DP and WDP plots. Iron sheets are buried around each plot to prevent runoff. Each treatment consists of six biological replicates.

Soils were collected in August 2013 and used for microbial analyses and soil variable measurements. Three soil cores with a diameter of 3.5 cm and a depth of 0 to 10 cm were randomly taken from each plot. The soil cores were mixed to make a composite sample, transported to the laboratory on ice, and immediately sieved with a 2-mm mesh to remove visible plant roots and stones. Soils for microbial analyses were stored at −80°C until DNA extraction. Soils for physiochemical measurements were stored at 4 or −20°C.

### Measurements of plant variables.

Plants were surveyed at the peak of the growing season in 2013, based on an area (0.5 m × 1.5 m) in the middle of each plot dedicated to plant survey. The area was divided into three subplots (0.5 m × 0.5 m) and plant species, and the corresponding coverage for each species were counted in each subplot. The average coverage of each species in the three subplots was calculated to represent the species coverage in the whole plot (2.2 m × 1.8 m). Plant diversity was calculated as follows using the Shannon index:

(1) $$H=-\overset{n}{\underset{i=1}{\sum }}\mathrm{pi}{\text{ log}}_{b}\mathrm{pi}\;$$

where pi is the coverage of species i divided by the coverage of total species.

Plants were harvested at the peak of the growing season in the year 2013 by completely clipping aboveground biomass to the ground level. Aboveground net primary production (ANPP) was the weight of living plants after harvesting and oven drying at 65°C to a constant weight. Belowground net primary production (BNPP) was measured by an ingrowth-core method at the end of the 2013 growing season (48). Briefly, we collected soil from cores with 50 cm in length and 5 cm in diameter, in which root biomass was removed in 2012. Roots in the soil cores were washed thoroughly to remove the attached soil. BNPP was calculated based on the weight of plant root after oven drying at 65°C to a constant weight.

### Measurements of soil variables and carbon fluxes.

Soil temperature and soil water content were automatically monitored on an hourly basis using 5-TM probe sensors at a depth of 5 cm and an EM-50 data logger (Meter, Inc., Pullman, WA). The soil pH was measured with a fresh soil/water ratio of 1:5 (vol/vol) by using an FE20 pH meter (FiveEasy; Giessen, Hessen Land, German). Soil total carbon (TC) and nitrogen (TN) were measured with a 2400 II CHN elemental analyzer (Perkin-Elmer, Inc., Boston, MA). Soil organic carbon (SOC) was calculated as the difference between TC and carbon bound to CaCO_3_. Dissolved organic carbon (DOC) and dissolved organic nitrogen (DON) were determined using a Multi N/C 3000 TOC analyzer (Analytik Jena AG, Thuringia, Germany). To determine nitrate and ammonium concentrations, 3 g of fresh soil was extracted with 2 M KCl for 1 h shaking, followed by filtering. The filtrate was measured with a continuous flow AutoAnalyzer 3 (Seal Analytical, Inc., Norderstedt, Schleswig-Holstein, Germany). Total phosphorus (TP) and soil available phosphorus (SAP) were measured using the molybdenum blue method with a UV-2550 UV-visible spectrophotometer (Shimadzu, Inc., Kyoto, Japan). Soil microbial biomass carbon (MBC) and microbial biomass nitrogen (MBN) were determined using the chloroform fumigation extraction method.

Soil CO_2_ and CH_4_ fluxes were measured using a static opaque chamber and Agilent 7890A gas chromatography (Agilent Technologies, Inc., Santa Clara, CA) as previously described (49). We measured CO_2_ and CH_4_ fluxes twice or three times per month on sunny days between 9:00 a.m. and 12:00 p.m. local time throughout 2013 to obtain average annual soil CO_2_ and CH_4_ fluxes. Briefly, soil CO_2_ fluxes and net ecosystem exchange (NEE) were measured by an LI-6400 infrared gas analyzer (LI-COR, Inc., Lincoln, NE). Before the measurement, square collars (0.4 m in length × 0.4 m in width × 0.1 m in height) were inserted at a depth of 5 cm. During each measurement, a transparent chamber (0.4 m in length × 0.4 m in width × 0.6 m in height; without the bottom) was placed on the collar and then sealed with water. A fan in each chamber was used to mix the air during sampling. After steady-state conditions were reached, six consecutive measurements of soil CO_2_ concentration, at 10-s intervals, were obtained over 1 min. The CO_2_ flux was then calculated from the slope of the linear regression of the measurements. Aboveground plants were removed when measuring soil CO_2_ flux, while aboveground plants were maintained when measuring NEE. The more negative value of NEE indicates greater carbon uptake from the atmosphere to the ecosystem. After the NEE measurement, we ventilated the chamber and covered it with an opaque cloth before measuring ecosystem respiration (ER). Gross primary productivity was then calculated by subtracting NEE from ER. CH_4_ gas samples were collected at 10-min intervals over 30 min using plastic syringes and then analyzed CH_4_ within 24 h using a gas chromatograph equipped with a flame ionization detector. The carrier gas was N_2_, and operating temperatures for the flame ionization detector were set at 250°C. Soil CH_4_ fluxes were calculated by the slope of linear regressions between gas concentration and sampling time (0, 10, 20, and 30 min after chamber closure).

### Ecosystem multifunctionality index.

Eight environmental variables were selected to calculate the ecosystem multifunctionality index (EMF): DOC, DON, SAP, MBC, ANPP, BNPP, and ER. Although the definition of EMF varies among individual studies, the majority of those studies have included variables associated with carbon, nitrogen, and phosphorus cycling, using associated enzyme activity (50) or soil nutrient content (24). DOC, DON, and SAP were selected because they are good surrogates for soil nutrients for soil microbes (41, 51, 52) and plants (24). ANPP, BNPP, and MBC represent the growth of plant and microbial communities. ER represents the conversion of organic carbon to CO_2_ in an ecosystem (53). We checked multicollinearity of selected variables using the *vif* function in the “car” package in R, and all variance inflation factors were <4, indicating low collinearity. To eliminate the effect of scale differences among seven environmental variables, we calculated the z-score transformation before using an averaging approach, in which there is no weight for each variable. The averaged multifunctionality (M*F_a_*) was calculated as follows:

(2) $$MF_{a}\;=\;\frac{1}{F}\overset{F}{\underset{i=1}{\sum }}g(f_{i})$$

where *F* is the number of measured functions, *f_i_* are the values of functions, and *g* is a function to standardize all the values of a function to an identical scale. The function *g* was calculated as follows:

(3) $$g=\;\frac{f_{i}\;-\;\bar{f}}{s}$$

where $\bar{f}$ is the mean value of *f_i_* and *s* is the standard deviation of *f_i_*.

### Amplicon sequencing experiments and raw data processing.

Soil DNA was extracted using the PowerSoi DNA isolation kit (MoBio Laboratories, Inc., Carlsbad, CA) according to the manufacturer’s instruction and then dissolved in nuclease-free water. The bacterial 16S rRNA gene and fungal internal transcribed spacer (ITS) region were sequenced using a phasing amplicon sequencing approach by a two-step PCR (54). The primer pair of 515-F (5′-GTGCCAGCMGCCGCGGTAA-3′) and 806-R (5′-GGACTACHVGGGTWTCTAAT-3′) was used to amplify the 16S rRNA gene. The primer pair of gITS7-F (5′-GTGARTCATCGARTCTTTG-3′) and ITS4-R (5′-TCCTCCGCTTATTGATATGC-3′) was used to amplify the ITS region. DNA template (5 μl) was mixed with PCR buffer (2.5 μl of 10 × PCR buffer, 1 μl of forward primer, 1 μl of reverse primer [10 μM], and 0.1 μl of AccuPrim Taq DNA polymerase high fidelity [Thermo Fisher Scientific, Inc., Carlsbad, CA]), brought to a volume of 25 μl with nuclease-free water and then amplified using the following programs. For 16S rRNA gene, there was an initial denaturation at 94°C for 1 min; followed by 10 cycles of 94°C for 20 s, 53°C for 25 s, and 68°C for 45 s (each); followed by a final extension at 68°C for 10 min. For the ITS region, there was an initial denaturation at 94°C for 3 min and then 14 cycles of 94°C for 30 s, 55°C for 0 s, and 68°C for 30 s (each), followed by a final extension at 68°C for 30 s. An aliquot (15 μl) of DNA product from the first-step PCR was then used as the template for the second-step PCR. The same buffer and amplification procedure was used except that 20 cycles were used for the 16S rRNA gene, and 26 cycles were used for the ITS region. Final PCR products were purified using Agencourt AMPure XP beads (Beckman Coulter, Inc., Brea, CA) and quantified by PicoGreen with a FLUOstar Optima plate reader (BMG Labtech, Inc., Ortenberg, Hesse, Germany). Purified DNA libraries were mixed with PhiX and then sequenced on a MiSeq sequencer (Illumina, Inc., San Diego, CA).

Raw sequence data were processed on a sequencing analysis pipeline (http://www.ou.edu/ieg/tools/data-analysis-pipeline) (55). Sequences were classified into OTUs using UPARSE with 97% similarity for the 16S rRNA gene and the ITS region and then rarefied by the minimum number of sequences detected across all samples (15,089 sequences for 16S rRNA gene and 10,570 sequences for the ITS region). OTU taxonomic classification was conducted using representative sequences from each OTU in RDP classifier with a confidence cutoff of 0.5 (56). Microbial MBC-normalized abundances of bacterial and fungal taxa were calculated by multiplying relative abundance of microbial taxa with corresponding MBC.

### Experiments with GeoChip and raw data processing.

Detailed descriptions of DNA labeling and hybridization to GeoChip 5.0 was described recently (22). Briefly, 0.6 μg of DNA labeled with fluorescent dye Cy-3 was hybridized with GeoChip 5.0 at 67°C plus 10% formamide in an Agilent hybridization oven for 24 h and scanned by a NimbleGen MS200 microarray scanner (Roche, Inc., Madison, WI). Raw GeoChip data were processed as follows: (i) poor-quality spots with a signal-to-noise ratio of <2.0 were removed, and (ii) functional gene probes that detected only one of six biological replicates were removed. The relative abundance of a functional gene was calculated by normalizing the total intensity for each sample. The measurement of microbial functional groups was independent of MBC since MBC was determined by the chloroform fumigation extraction method, while functional genes were revealed by GeoChip analysis. Since the effect of soil microbes on ecosystem functions depends on microbial biomass (57), we calculated the abundances of microbial functional genes by multiplying the relative abundance of functional genes by the corresponding MBC to standardize samples across different microbial biomasses.

### Statistical analyses.

The microbial taxonomic or functional α*-*diversity was calculated by using the Shannon index:

(4) $$H=-\overset{n}{\underset{i=1}{\sum }}\mathrm{pi}\;\log_{b}\mathrm{pi}\;$$

where pi is the percentage of species i to total species.

The dissimilarity of within-group samples was calculated as mean Bray-Curtis weighted dissimilarities between pairs of replicates in a group. Since the distribution of Bray-Curtis weighted dissimilarities did not fit the normal model, the Kruskal-Wallis H-rank sum test was used to conduct *post hoc* tests using the *Kruskal.test* and *pairwise.wilcox.test* functions in the “stats” package.

Linear mixed-effects models (LMEM) were fitted to reveal the effect sizes of variables on soil carbon fluxes and NEE. The model structure was set as CO_2_ ∼ ANPP + SOC + gene abundance + (1|block), in which different variables were used as fixed effects, and the block was used as a random intercept effect. Coefficients were regarded to be significant if *P* < 0.050. The *r*^2^ values of the model considering the fixed effect and random effect were used to explain variations of soil carbon fluxes. Similarly, we used LMEM to assess the effects of treatments on plants, soil variables, and microbial abundances, considering the block effect. The model structure was set as *y* ∼ warming × precipitation alteration + (1|block), where *y* is the plant or soil variable or the microbial abundance tested. The *lmer* function in the “lme4” package was used to run LMEM. The statistical significance of the coefficient in LMEM was based on type-II analysis-of-variance tables calculated by the *Anova* function in the “car” package. A *post hoc* Turkey’s honestly significant difference test was conducted using *glht* and *cld* function in the “multicomp” package. The Pearson correlation test was conducted with the *cor.test* function in the “stats” package. *P* values were adjusted by using the Bonferroni algorithm with the *p.adjust* function in the “stats” package.

Multivariate analyses were used to link soil or plant variables to microbial community compositions. PerMANOVA based on weighted Bray-Curtis distance was used to detect the main and interactive effects of warming and precipitation alteration on microbial communities with the block as a random effect. To this end, a distance matrix was extracted from the overall microbial community composition. *F*-statistic and *P* values of PerMANOVA were obtained by partition variance and observation permutations. The plant Bray-Curtis distance matrix was based on a multivariate (36 × 3) matrix comprised of three selected plant variables of ANPP, BNPP, and plant Shannon diversity in 36 plots. The model structure was set as distance ∼ warming × precipitation alteration+ (1/block). The *adonis* function of the “vegan” package was used to run PerMANOVA for microbial community data. Partial Mantel tests were conducted to detect the linkages between environmental variables and microbial communities and to determine the influences of microbes, plants, and soil variables on carbon fluxes. Specifically, dissimilarity matrices were calculated with weighted Bray-Curtis distance. The linkages of microbial functional composition of recalcitrant carbon degradation with carbon fluxes were judged by *r* values. In partial Mantel tests, selected plant variables of ANPP, BNPP, and plant Shannon diversity were used. We calculated plant dissimilarity matrix using weighted Bray-Curtis distance based on a multivariate (36 × 3) matrix comprised of three plant variables derived from 36 plots. As an extension of partial Mantel tests, multiple regression on distance matrices (MRM) could contain more independent variables of matrix type. The method was used to determine the influence of selected plant variables, soil variables, and microbial functional composition on carbon fluxes. Selected plant variables of ANPP, BNPP, and plant Shannon diversity were used in MRM. MRM was conducted by using the *MRM* function in the “ecodist” package. The procrustes analysis was conducted to depict correlations between microbial functional composition and taxonomic composition using *procrustes* and *protest* function in the “vegan” package. These analyses were carried out in R-studio v3.5.2.

### Data availability.

GeoChip data can be accessed through the URL http://www.ou.edu/ieg/publications/datasets. MiSeq data are available in the NCBI SRA database with accession number PRJNA540144.
