# Supplementary material for: Microbial Functional Responses Explain Alpine Soil Carbon Fluxes under Future Climate Scenarios
Source: mBio. 2021 Feb 23;12(1):e00761-20. doi: 10.1128/mBio.00761-20 (PMC8545085; doi:10.1128/mBio.00761-20)
Supplement: TABLE S1 [file mbio.00761-20-st001.docx]

**Table S1.** **Environmental variables**

| Environmental variables | Control | Wetting | Drying | Warming | Warming + Wetting | Warming + Drying |
| --- | --- | --- | --- | --- | --- | --- |
| Clay (%)^a^ | 65.33±4.36a^b^ | 66.97±3.94ab | 72.40±2.70ab | 60.32±6.70a | 63.86±4.95a | 78.67±2.32b |
| Silt (%) | 11.85±0.87a | 12.09±0.63a | 10.48±1.35a | 14.68±2.65a | 12.16±1.34a | 10.93±0.73a |
| Sandy (%) | 22.82±3.61b | 20.94±3.33b | 17.11±2.81ab | 25.01±4.25b | 23.98±3.78b | 10.39±2.70a |
| pH | 7.85±0.07ab | 7.86±0.03b | 7.89±0.01b | 7.91±0.01b | 7.77±0.07a | 7.90±0.02b |
| SWC (g kg^-1^) | 32.48±1.50b | 35.91±2.00b | 28.30±3.90ab | 28.40±2.15ab | 34.48±2.09b | 22.21±1.03a |
| ST (^o^C) | 4.77±0.30a | 4.82±0.50a | 4.96±0.28ab | 6.67±0.47c | 6.36±0.11bc | 7.38±0.57c |
| TC (g kg^-1^) | 78.18±0.95a | 75.78±3.95a | 81.35±7.45a | 75.05±7.59a | 79.50±2.48a | 79.90±4.82a |
| TN (g kg^-1^) | 5.75±0.28a | 5.55±0.34a | 6.25±0.60a | 5.55±0.79a | 5.85±0.13a | 5.88±0.37a |
| TP (g kg^-1^) | 0.79±0.05ab | 0.82±0.03ab | 0.87±0.05b | 0.75±0.06ab | 0.83±0.02ab | 0.71±0.04a |
| SOC (g kg^-1^) | 63.10±1.04a | 62.77±3.86a | 67.09±4.57a | 65.39±3.37a | 65.02±1.79a | 64.42±3.90a |
| NH_4_^+^ (mg kg^-1^) | 27.29±4.20a | 26.32±0.34a | 30.50±3.13a | 23.73±2.05a | 21.30±3.59a | 28.18±3.74a |
| NO_3_^-^ (mg kg^-1^) | 10.27±0.47b | 8.61±0.33a | 11.61±0.35cd | 10.47±0.41bc | 10.11±0.35b | 12.41±0.10d |
| SAP (mg kg^-1^) | 7.68±0.17a | 7.43±0.52a | 6.83±0.41a | 6.72±0.41a | 7.60±0.20a | 6.60±0.32a |
| DOC (mg kg^-1^) | 280.31±6.07bd | 245.69±11.58ab | 304.60±10.41cd | 268.92±8.47abc | 240.28±10.49a | 311.65±5.75d |
| DON (mg kg^-1^) | 33.03±3.28a | 29.01±2.56a | 40.67±4.03a | 31.11±1.42a | 30.94±4.00a | 37.33±4.81a |
| MBC (mg kg^-1^) | 656.79±34.20bc | 710.64±45.61c | 567.29±36.49ab | 552.57±19.10ab | 688.57±36.11bc | 477.64±25.37a |
| MBN (mg kg^-1^) | 61.40±1.92bc | 73.79±2.98c | 55.70±5.72b | 59.93±5.21b | 66.24±3.07bc | 42.93±0.72a |
| CO_2_ (mg m^-2^ h^-1^) | 647.96±48.93abc | 710.36±28.40cd | 587.56±23.87ab | 635.76±42.97ac | 772.54±46.49d | 545.66±36.24a |
| CH_4_ (μg m^-2^ h^-1^) | -56.05±5.70bcd | -42.76±2.76d | -75.71±5.29ab | -69.74±4.25ac | -54.84±8.29cd | -84.97±0.32a |
| N_2_O (μg m^-2^ h^-1^) | 11.59±4.43a | -2.01±0.83a | 2.94±7.89a | 3.69±1.12a | 0.26±0.65a | -0.76±4.24a |
| Grass ANPP (g m^-2^) | 175.65±33.36a | 221.96±14.67ab | 188±23.94a | 226.07±9.59ab | 322.35±64.41b | 152.73±11.71a |
| Sedge ANPP (g m^-2^) | 37.60±5.78ab | 51.16±19.70b | 18.59±4.81a | 15.14±6.83a | 32.70±2.59ab | 12.36±3.16a |
| Forb ANPP (g m^-2^) | 146.26±3.30c | 104.08±20.77ac | 80.38±3.57a | 93.99±14.06ab | 134.75±2.96bc | 58.92±11.08a |
| Other ANPP (g m^-2^) | 0.00±0.00a | 0.00±0.00a | 0.69±0.69a | 0.15±0.15a | 0.03±0.03a | 0.00±0.00a |
| ANPP (g m^-2^) | 359.51±38.45bc | 377.20±13.40bc | 287.65±22.91ab | 335.35±11.66ab | 489.82±62.14c | 224.01±17.29a |
| Total plant biomass | 1088.11±125.49a | 993.82±26.50a | 995.78±102.15a | 998.48±55.82a | 1109.88±24.95a | 943.96±123.53a |
| Root/shoot ratio | 2.00±0.05bc | 1.63±0.05c | 2.44±0.08ab | 1.98±0.24bc | 1.32±0.25c | 3.23±0.51a |
| Grass richness | 6.50±0.65a | 6.25±0.48a | 5.75±0.63a | 5.00±0.58a | 7.00±0.58a | 4.75±0.48a |
| Sedge richness | 2.75±0.25ab | 2.75±0.25ab | 2.00±0.00a | 2.75±0.25ab | 3.00±0.00b | 2.75±0.25ab |
| Forb richness | 21.00±1.08bc | 24.00±1.35c | 20.25±0.63ac | 19.00±1.22ab | 23.00±0.00c | 17.00±0.82a |
| Other plant richness | 0.25±0.25a | 0.00±0.00a | 0.25±0.25a | 0.00±0.00a | 0.25±0.25a | 0.00±0.00a |
| Plant richness | 30.50±1.50bc | 33.00±1.73c | 28.25±1.31ac | 26.75±1.38ab | 33.25±0.75c | 24.50±0.65a |
| Plant diversity | 1.72±0.11a | 1.55±0.15a | 1.49±0.18a | 1.45±0.15a | 1.66±0.20a | 1.12±0.13a |
| BNPP (g m^-2^) | 726.73±88.42a | 616.62±16.94a | 707.89±79.09a | 662.85±63.33a | 595.44±75.55a | 719.96±118.79a |
| GPP (μmol CO_2_ m^-2^ s^-1^) | 16.38±1.40a | 20.27±0.73b | 17.11±0.51ab | 19.72±1.27b | 24.04±0.35c | 16.21±1.18a |
| NEE (μmol CO_2_ m^-2^ s^-1^) | -10.08±0.74cd | -13.04±0.78b | -10.64±0.62cd | -11.78±1.17bc | -15.82±0.34a | -9.30±1.09d |
| ER (μmol CO_2_ m^-2^ s^-1^) | 6.30±0.72a | 7.23±0.06ac | 6.47±0.67a | 7.94±0.37bc | 8.22±0.12c | 6.91±0.21ab |
| EMF | 0.19±0.18bc | -0.11±0.04ac | -0.04±0.14ac | -0.24±0.12ab | 0.34±0.10c | -0.36±0.07a |

^a^Abbreviations: Clay, soil clay content, Silt, soil silt content, Sandy, soil sandy content; SWC, soil water content; ST, soil temperature; TC, soil total carbon; TN, soil total nitrogen; TP, soil total phosphorus; SOC, soil organic carbon; SAP, soil available phosphorus; DOC, dissolved organic carbon; DON, dissolved organic nitrogen; MBC, microbial biomass carbon; MBN, microbial biomass nitrogen; CO_2_, soil CO_2_ flux; CH_4_, soil CH_4_ flux; N_2_O, soil N_2_O flux; Grass ANPP, the aboveground net primary production of grass; Sedge ANPP, the aboveground net primary production of sedge; Forb ANPP, the aboveground net primary production of forb; Other ANPP, the aboveground net primary production of other functional groups of plant; ANPP, the aboveground net primary production of plant community; Total plant biomass, the sum of aboveground and belowground net primary production; Root/shoot ratio, the ratio of BNPP to ANPP; the Grass richness, species number of grass; Sedge richness, species number of sedge; Forb richness, species number of forb; Other plant richness, species number of other functional groups of plants; Plant richness, the species numbers of plant community; Plant diversity, the α-diversity of plant community based on Shannon index; BNPP, belowground net primary production; NEE, net ecosystem exchange; ER, ecosystem respiration; GPP, gross primary productivity; EMF, ecosystem multifunctionality.

^b^Mean values (± s.e.) are shown. The letters were calculated by post-hoc Tukey’s honest significant difference test of a linear mixed-effects model. Different alphabetic letters indicate significant difference (*P* < 0.050).
