# Supplementary material for: Microbial Functional Responses Explain Alpine Soil Carbon Fluxes under Future Climate Scenarios
Source: mBio. 2021 Feb 23;12(1):e00761-20. doi: 10.1128/mBio.00761-20 (PMC8545085; doi:10.1128/mBio.00761-20)
Supplement: TABLE S2 [file mbio.00761-20-st002.docx]

**Table S2.** **Treatment effects on environmental variables and microbial communities^a^**

| Variables | | Temperature | Precipitation | Interaction |
| --- | --- | --- | --- | --- |
| Environmental variables | Clay (%)^b^ | 0.01 | 13.40** | 3.36 |
|  | Silt (%) | 3.01 | 2.06 | 0.84 |
|  | Sandy (%) | 0.93 | 14.86*** | 5.57 |
|  | pH | 0.41 | 4.94* | 4.41* |
|  | SWC (g kg^-1^) | 5.95* | 14.02*** | 1.20 |
|  | ST (^o^C) | 68.11*** | 1.73 | 0.08 |
|  | TC (g kg^-1^) | 0.01 | 0.77 | 0.57 |
|  | TN (g kg^-1^) | 0.16 | 1.25 | 0.76 |
|  | TP (g kg^-1^) | 5.95* | 14.03*** | 1.20 |
|  | SOC (g kg^-1^) | 0.12 | 0.39 | 0.81 |
|  | NH_4_^+^ (mg kg^-1^) | 7.89** | 6.38** | 0.36 |
|  | NO_3_^-^ (mg kg^-1^) | 8.59** | 38.08*** | 1.29 |
|  | SAP (mg kg^-1^) | 2.93 | 3.80* | 2.18 |
|  | DOC (mg kg^-1^) | 0.11 | 40.86*** | 2.31 |
|  | DON (mg kg^-1^) | 0.01 | 2.70 | 0.20 |
|  | CO_2_ (mg m^-2^ h^-1^) | 1.51 | 22.83*** | 1.95 |
|  | CH_4_ (μg m^-2^ h^-1^) | 12.34** | 28.21*** | 0.44 |
|  | N_2_O (μg m^-2^ h^-1^) | 3.07 | 0.36 | 2.48 |
|  | Grass ANPP (g m^-2^) | 1.00 | 5.07* | 2.29 |
|  | Sedge ANPP (g m^-2^) | 6.10* | 6.92** | 0.38 |
|  | Forb ANPP (g m^-2^) | 4.68* | 14.94*** | 5.33* |
|  | Other ANPP (g m^-2^) | 0.57 | 0.81 | 1.34 |
|  | ANPP (g m^-2^) | 0.34 | 20.28*** | 4.46* |
|  | Total plant biomass | 0.58 | 18.46*** | 4.05* |
|  | Root/shoot ratio | 0.27 | 15.09*** | 1.88 |
|  | Grass richness | 5.01* | 5.32* | 4.10* |
|  | Sedge richness | 8.57* | 6.96* | 3.75* |
|  | Forb richness | 9.17** | 12.13*** | 0.82 |
|  | Other plant richness | 1.19 | 0.01 | 4.76* |
|  | Plant richness | 8.03** | 14.60*** | 2.24 |
|  | Plant diversity | 3.34 | 3.89* | 1.65 |
|  | BNPP (g m^-2^) | 1.09 | 0.79 | 0.86 |
|  | NEE (μmol CO_2_ m^-2^ s^-1^) | 2.43 | 18.45*** | 4.41* |
|  | ER (μmol CO_2_ m^-2^ s^-1^) | 14.54*** | 8.13*** | 0.76 |
|  | GPP (μmol CO_2_ m^-2^ s^-1^) | 5.77* | 15.36*** | 2.73 |
|  | EMF | 3.24 | 6.36** | 4.22* |
| Microbial biomass | MBC (mg kg^-1^) | 8.76** | 16.89*** | 1.52 |
|  | MBN (mg kg^-1^) | 6.35* | 26.46*** | 1.72 |
| Microbial functional communities | Overall functional community | 0.71 | 2.67* | 1.14 |
|  | *amyA* | 0.79 | 2.37* | 1.04 |
|  | *amyX* | 0.74 | 2.54 | 5.91* |
|  | *apu* | 0.10 | 1.78 | 0.43 |
|  | *cda* | 1.10 | 2.50** | 1.14 |
|  | *glucoamylase* | 1.02 | 2.35** | 1.04 |
|  | *isopullulanase* | 0.13 | 1.01 | 0.93 |
|  | *nplT* | 1.80 | 3.16*** | 2.86*** |
|  | *pulA* | 1.02 | 2.82** | 1.47 |
|  | *ara* | 0.74 | 2.95* | 1.15 |
|  | *mannanase* | 1.09 | 3.39* | 1.29 |
|  | *xylA* | 0.83 | 1.78* | 0.97 |
|  | *xylanase* | 0.73 | 1.80* | 1.08 |
|  | *axe* | 0.72 | 2.48* | 1.12 |
|  | *cellobiase* | 1.12 | 1.88* | 1.26 |
|  | *GH7 cellulase* | 1.03 | 6.13** | 0.82 |
|  | *endoglucanase* | 0.97 | 2.32* | 1.04 |
|  | *exoglucanase* | 1.03 | 2.03* | 0.96 |
|  | *acetylglucosaminidase* | 0.88 | 2.99* | 1.26 |
|  | *chitin deacetylase* | 0.98 | 2.48* | 1.43 |
|  | *chitinase* | 0.79 | 2.53* | 1.11 |
|  | *glx* | 0.94 | 3.16** | 1.40 |
|  | *ligninase* | 0.58 | 1.92* | 2.12* |
|  | *mnp* | 0.22 | 1.93 | 0.83 |
|  | *phenol oxidase* | 1.19 | 2.49** | 1.42 |
|  | *fwdB* | 1.57 | 2.68 | 0.66 |
|  | *ftr* | 0.47 | 4.35** | 1.06 |
|  | *hmd* | 2.02 | 2.29* | 2.44 |
|  | *mch* | 1.24 | 3.42* | 1.02 |
|  | *mtrH* | 2.68 | 5.90*** | 1.82 |
|  | *mer* | 0.84 | 2.19* | 1.66 |
|  | *acs* | 3.14 | 0.56 | 0.96 |
|  | *mtaB* | 0.57 | 5.34*** | 3.00* |
|  | *mtmB* | 2.44 | 6.97** | 1.86 |
|  | *mtbC* | 0.20 | 1.76 | 1.22 |
|  | *mcrA* | 0.66 | 3.20* | 0.92 |
|  | *MT2* | 0.47 | 1.41 | 2.59** |
|  | *hdrB* | 1.46 | 3.54* | 1.22 |
|  | *mmoX* | 0.68 | 1.86 | 2.25* |
|  | *pmoA* | 1.02 | 2.82** | 1.47 |
| Bacterial communities | Overall bacterial community | 1.27 | 1.58* | 1.04 |
|  | Unclassified | 1.04 | 1.07 | 1.05 |
|  | *Alphaproteobacteria* | 1.15 | 1.30 | 1.10 |
|  | *Deltaproteobacteria* | 1.18 | 1.58 | 1.16 |
|  | *Gammaproteobacteria* | 0.88 | 3.23 | 1.16 |
|  | *Betaproteobacteria* | 1.98 | 2.94 | 1.48 |
|  | *Acidobacteria* | 0.91 | 1.27 | 1.06 |
|  | *Actinobacteria* | 1.73 | 1.26 | 0.80 |
|  | *Planctomycetes* | 1.12 | 1.04 | 0.96 |
|  | *Gemmatimonadetes* | 0.84* | 1.11 | 1.07 |
|  | *Bacteroidetes* | 1.60 | 2.40** | 1.37 |
|  | *Firmicutes* | 0.43 | 0.70 | 0.75 |
|  | *Chloroflexi* | 0.99 | 1.17 | 1.09 |
|  | *Verrucomicrobia* | 1.29 | 1.54 | 1.08 |
|  | *Candidate division WPS 1* | 0.71 | 1.12 | 0.96 |
|  | *Armatimonadetes* | 1.10 | 2.23* | 1.54 |
|  | *Thaumarchaeota* | 1.04 | 2.83* | 1.00 |
|  | *Latescibacteria* | 0.34 | 1.22 | 1.07 |
|  | *Nitrospirae* | 1.00 | 0.66 | 1.73 |
| Fungal communities | Overall fungal community | 0.94 | 1.24* | 1.18 |
|  | *Ascomycota* | 0.98 | 1.20 | 1.19 |
|  | *Basidiomycota* | 0.99 | 1.31 | 1.16 |
|  | Unclassified | 0.90 | 0.92 | 0.97 |
|  | *Zygomycota* | 0.89 | 1.36 | 1.26 |
|  | *Chytridiomycota* | 0.56 | 1.38 | 1.65* |
|  | *Glomeromycota* | 0.95 | 1.24* | 1.26* |

^a^ANOVA based on the linear mixed-effects model was used to test treatment effects on environmental variables. PerMANOVA is based on the linear mixed-effects model with a weighted Bray-Curtis dissimilarity matrix to test treatment effects on microbial communities. *F*-statistics are shown. Significance is indicated by **P* < 0.050, ***P* < 0.010, ****P* < 0.001.

^b^Abbreviations: Clay, soil clay content, Silt, soil silt content, Sandy, soil sandy content; SWC, soil water content; ST, soil temperature; TC, soil total carbon; TN, soil total nitrogen; TP, soil total phosphorus; SOC, soil organic carbon; SAP, soil available phosphorus; DOC, dissolved organic carbon; DON, dissolved organic nitrogen; CO_2_, soil CO_2_ flux; CH_4_, soil CH_4_ flux; N_2_O, soil N_2_O flux; Grass ANPP, the aboveground net primary production of grass; Sedge ANPP, the aboveground net primary production of sedge; Forb ANPP, the aboveground net primary production of forb; Other ANPP, the aboveground net primary production of other functional groups of plant; ANPP, the aboveground net primary production of plant community; Total plant biomass, the sum of aboveground and belowground net primary production; Root/shoot ratio, the ratio of BNPP to ANPP; the Grass richness, species number of grass; Sedge richness, species number of sedge; Forb richness, species number of forb; Other plant richness, species number of other functional groups of plants; Plant richness, the species numbers of plant community; Plant diversity, the α-diversity of plant community based on Shannon index; BNPP, belowground net primary production; NEE, net ecosystem exchange; ER, ecosystem respiration; GPP, gross primary productivity; EMF, ecosystem multifunctionality; MBC, microbial biomass carbon; MBN, microbial biomass nitrogen.
