# Supplementary material for: Microbial Functional Responses Explain Alpine Soil Carbon Fluxes under Future Climate Scenarios
Source: mBio. 2021 Feb 23;12(1):e00761-20. doi: 10.1128/mBio.00761-20 (PMC8545085; doi:10.1128/mBio.00761-20)
Supplement: FIG S1 [file mbio.00761-20-sf001.docx]

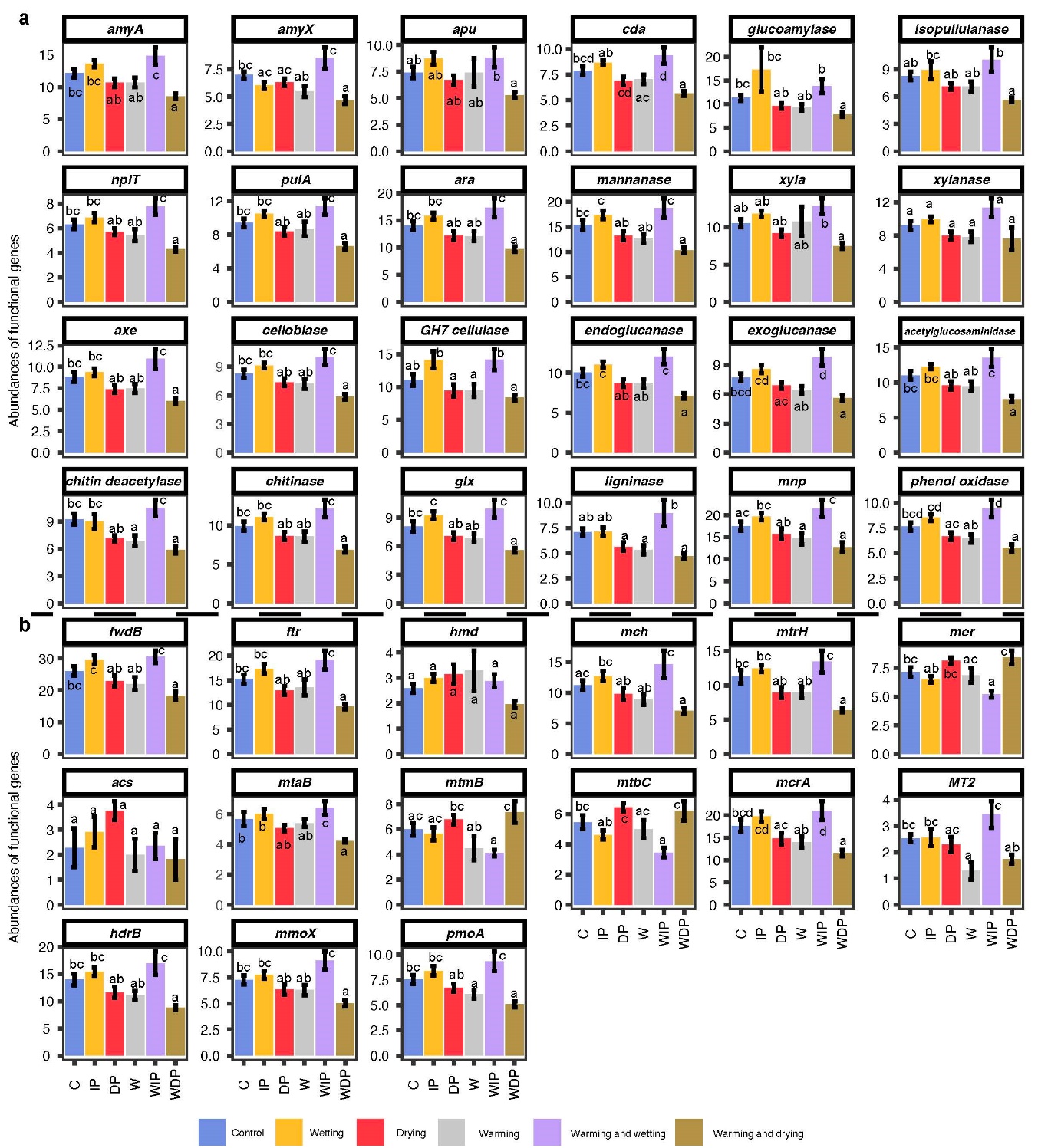


**Fig. S1 The MBC-normalized abundances of microbial functional genes associated with carbon degradation (a) and methane cycling (b).** Functional genes associated with starch degradation (*amyA*, *amyX*, *apu*, *cda*, *glucoamylase*, *isopullulanase*, *nplT,* and *pulA*); hemicellulose degradation (*ara*, *mannanase*, *xylA,* and *xylanase*); cellulose degradation (*axe*, *cellobiase*, *GH7 cellulase*, *endoglucanase,* and *exoglucanase*); chitin degradation (*acetylglucosaminidase*, *chitin deacetylase,* and *chitinase*); lignin degradation (*glx*, *ligninase*, *mnp,* and *phenol oxidase*); methanogenesis (*fwdB*, *ftr*, *hmd*, *mch*, *mtrH*, *mer,* *acs, mtaB*, *mtmB, mtbC, mcrA, MT2, and hdrB*) and methane oxidation (*mmoX* and *pmoA*) are shown. Different alphabets on the bars were calculated by post-hoc Tukey’s honest significant difference test with a linear mixed-effects model (*P* < 0.050).
