# Supplementary material for: Microbial Functional Responses Explain Alpine Soil Carbon Fluxes under Future Climate Scenarios
Source: mBio. 2021 Feb 23;12(1):e00761-20. doi: 10.1128/mBio.00761-20 (PMC8545085; doi:10.1128/mBio.00761-20)
Supplement: FIG S2 [file mbio.00761-20-sf002.docx]

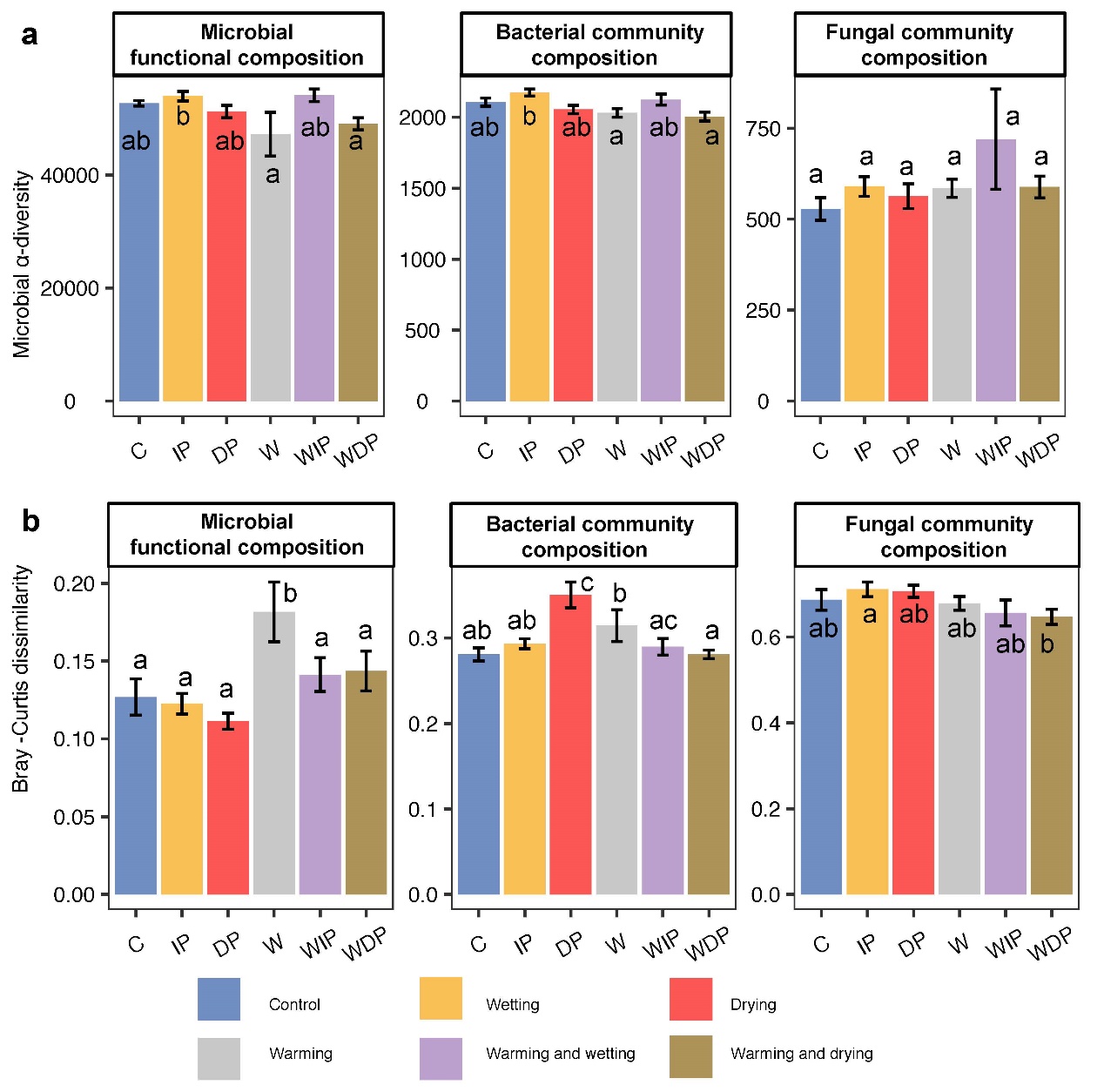


**Fig. S2 α-diversity (a) and**  **mean Bray-Curtis weighted dissimilarities (b) of soil microbial communities under different treatments.** Different alphabets on the bars associated with α-diversity were calculated by post-hoc Tukey’s honest significant difference test with a linear mixed-effects model (*P* < 0.050). Different alphabets on the bars associated with within-group dissimilarities were calculated by Kruskal-Wallis H-test (*P* < 0.050). The dissimilarity of within-group samples was calculated as mean Bray-Curtis weighted dissimilarities between pairs of replicates in a group.
