# Supplementary material for: Microbial Functional Responses Explain Alpine Soil Carbon Fluxes under Future Climate Scenarios
Source: mBio. 2021 Feb 23;12(1):e00761-20. doi: 10.1128/mBio.00761-20 (PMC8545085; doi:10.1128/mBio.00761-20)
Supplement: TABLE S3 [file mbio.00761-20-st003.docx]

**Table S3.** **Correlations between carbon fluxes and relative abundances of microbial functional genes**

| Carbon fluxes | Category | Subcategory | Gene | *r* | *P* |
| --- | --- | --- | --- | --- | --- |
| CO_2_^a^ | Carbon degradation | Starch | *amyA* | 0.65 | 0.001**^b^ |
|  | Carbon degradation | Starch | *amyX* | 0.30 | 0.588 |
|  | Carbon degradation | Starch | *apu* | 0.29 | 0.588 |
|  | Carbon degradation | Starch | *cda* | 0.63 | 0.002** |
|  | Carbon degradation | Starch | *glucoamylase* | 0.23 | 0.749 |
|  | Carbon degradation | Starch | *isopullulanase* | 0.48 | 0.035* |
|  | Carbon degradation | Starch | *nplT* | 0.55 | 0.011* |
|  | Carbon degradation | Starch | *pulA* | 0.61 | 0.003** |
|  | Carbon degradation | Hemicellulose | *ara* | 0.65 | 0.001** |
|  | Carbon degradation | Hemicellulose | *mannanase* | 0.68 | < 0.001*** |
|  | Carbon degradation | Hemicellulose | *xylA* | 0.62 | 0.002** |
|  | Carbon degradation | Hemicellulose | *xylanase* | 0.35 | 0.320 |
|  | Carbon degradation | Cellulose | *axe* | 0.66 | 0.001** |
|  | Carbon degradation | Cellulose | *cellobiase* | 0.61 | 0.003** |
|  | Carbon degradation | Cellulose | *GH7 cellulase* | 0.62 | 0.003** |
|  | Carbon degradation | Cellulose | *endoglucanase* | 0.64 | 0.002** |
|  | Carbon degradation | Cellulose | *exoglucanase* | 0.61 | 0.003** |
|  | Carbon degradation | Chitin | *acetylglucosaminidase* | 0.64 | 0.002** |
|  | Carbon degradation | Chitin | *chitin deacetylase* | 0.59 | 0.004** |
|  | Carbon degradation | Chitin | *chitinase* | 0.64 | 0.002** |
|  | Carbon degradation | Lignin | *glx* | 0.63 | 0.002** |
|  | Carbon degradation | Lignin | *ligninase* | 0.55 | 0.011* |
|  | Carbon degradation | Lignin | *mnp* | 0.53 | 0.014* |
|  | Carbon degradation | Lignin | *phenol oxidase* | 0.61 | 0.003** |
| CH_4_ | Methane | Methanogenesis | *fwdB* | 0.48 | 0.050 |
|  | Methane | Methanogenesis | *ftr* | 0.64 | 0.001** |
|  | Methane | Methanogenesis | *hmd* | 0.21 | 0.999 |
|  | Methane | Methanogenesis | *mch* | 0.48 | 0.050 |
|  | Methane | Methanogenesis | *mtrH* | 0.66 | < 0.001*** |
|  | Methane | Methanogenesis | *mer* | 0.42 | 0.098 |
|  | Methane | Methanogenesis | *acs* | 0.03 | 0.999 |
|  | Methane | Methanogenesis | *mtaB* | 0.13 | 0.999 |
|  | Methane | Methanogenesis | *mtmB* | 0.05 | 0.999 |
|  | Methane | Methanogenesis | *mtbC* | 0.49 | 0.039* |
|  | Methane | Methanogenesis | *mcrA* | 0.56 | 0.008** |
|  | Methane | Methanogenesis | *MT2* | 0.21 | 0.999 |
|  | Methane | Methanogenesis | *hdrB* | 0.57 | 0.006** |
|  | Methane | Methanotrophy | *mmoX* | 0.45 | 0.078 |
|  | Methane | Methanotrophy | *pmoA* | 0.42 | 0.100 |

^a^Abbreviations: CO_2_, soil CO_2_ flux; CH_4_, soil CH_4_ flux.

^b^Significance is indicated by **P* < 0.050; ***P* < 0.010; ****P* < 0.001
