# Supplementary material for: Microbial Functional Responses Explain Alpine Soil Carbon Fluxes under Future Climate Scenarios
Source: mBio. 2021 Feb 23;12(1):e00761-20. doi: 10.1128/mBio.00761-20 (PMC8545085; doi:10.1128/mBio.00761-20)
Supplement: TABLE S4 [file mbio.00761-20-st004.docx]

**Table S4.** **Correlations between carbon fluxes and the MBC-normalized abundances of major bacterial and fungal taxa**

|  |  | CO_2_^a^ | |  | CH_4_ | |  | NEE | |
| --- | --- | --- | --- | --- | --- | --- | --- | --- | --- |
|  |  | *r* | *P* |  | *r* | *P* |  | *r* | *P* |
| Bacterial phylum | *Acidobacteria* | 0.27 | 0.366 |  | 0.44 | 0.046*^b^ |  | -0.39 | 0.100 |
|  | *Actinobacteria* | 0.02 | 0.935 |  | 0.17 | 0.564 |  | -0.04 | 0.908 |
|  | *Bacteroidetes* | 0.47 | 0.024* |  | 0.55 | < 0.001*** |  | -0.35 | 0.169 |
|  | *Candidate division WPS 1* | 0.11 | 0.736 |  | 0.14 | 0.635 |  | 0.05 | 0.902 |
|  | *Firmicutes* | -0.10 | 0.736 |  | -0.04 | 0.908 |  | 0.12 | 0.705 |
|  | *Gemmatimonadetes* | -0.05 | 0.898 |  | 0.08 | 0.793 |  | -0.32 | 0.221 |
|  | *Latescibacteria* | 0.16 | 0.594 |  | 0.23 | 0.434 |  | -0.56 | < 0.001*** |
|  | *Nitrospirae* | -0.18 | 0.564 |  | -0.05 | 0.902 |  | -0.19 | 0.550 |
|  | *Proteobacteria* | 0.57 | < 0.001*** | | 0.71 | < 0.001*** |  | -0.53 | 0.013* |
|  | *Verrucomicrobia* | 0.23 | 0.434 |  | 0.17 | 0.564 |  | -0.17 | 0.564 |
|  | *Agromyces* | -0.06 | 0.875 |  | 0.12 | 0.685 |  | 0.04 | 0.908 |
|  | *Asanoa* | 0.15 | 0.632 |  | 0.19 | 0.550 |  | 0.00 | 0.987 |
|  | *Blastococcus* | 0.06 | 0.882 |  | 0.19 | 0.550 |  | 0.02 | 0.947 |
|  | *Bradyrhizobium* | 0.26 | 0.366 |  | 0.42 | 0.068 |  | -0.25 | 0.398 |
| Bacterial genus | *Nitrosospira* | 0.32 | 0.221 |  | 0.48 | 0.036* |  | -0.44 | 0.052 |
|  | *Oryzihumus* | 0.22 | 0.465 |  | 0.33 | 0.210 |  | -0.04 | 0.908 |
|  | *Phyllobacterium* | -0.12 | 0.692 |  | 0.30 | 0.249 |  | -0.04 | 0.908 |
|  | *Pseudonocardia* | 0.33 | 0.210 |  | 0.41 | 0.077 |  | -0.16 | 0.594 |
|  | *Rhizobacter* | 0.48 | 0.024* |  | 0.52 | 0.013* |  | -0.48 | 0.024* |
|  | *Variovorax* | 0.54 | 0.013* |  | 0.51 | 0.013* |  | -0.38 | 0.115 |
|  | *Ascomycota* | 0.26 | 0.366 |  | 0.23 | 0.434 |  | -0.44 | 0.046* |
|  | *Basidiomycota* | 0.17 | 0.564 |  | 0.30 | 0.257 |  | -0.02 | 0.940 |
| Fungal phylum | *Chytridiomycota* | -0.09 | 0.763 |  | -0.13 | 0.661 |  | 0.13 | 0.661 |
|  | *Glomeromycota* | -0.09 | 0.763 |  | -0.18 | 0.564 |  | 0.17 | 0.564 |
|  | *Zygomycota* | 0.29 | 0.302 |  | 0.47 | 0.024* |  | -0.30 | 0.249 |
| Fungal genus | *Chalara* | 0.23 | 0.434 |  | 0.24 | 0.404 |  | -0.26 | 0.366 |
|  | *Davidiella* | -0.16 | 0.594 |  | -0.10 | 0.750 |  | -0.19 | 0.564 |
|  | *Eurotiales* | 0.12 | 0.705 |  | -0.11 | 0.705 |  | -0.22 | 0.447 |
|  | *Eurotiomycetes* | 0.07 | 0.839 |  | 0.13 | 0.661 |  | 0.10 | 0.761 |
|  | *Herpotrichiellaceae* | -0.04 | 0.908 |  | -0.02 | 0.940 |  | -0.17 | 0.564 |
|  | *Hygrocybe* | -0.03 | 0.935 |  | 0.18 | 0.564 |  | 0.13 | 0.661 |
|  | *Leptosphaeria* | 0.25 | 0.384 |  | 0.32 | 0.221 |  | -0.02 | 0.935 |
|  | *Ramariopsis* | 0.36 | 0.158 |  | 0.05 | 0.908 |  | -0.41 | 0.076 |
|  | *Tetracladium* | 0.16 | 0.594 |  | 0.24 | 0.415 |  | -0.26 | 0.366 |
|  | *Tricholoma* | -0.01 | 0.978 |  | -0.15 | 0.632 |  | 0.09 | 0.763 |

^a^Abbreviations: CO_2_, soil CO_2_ flux; CH_4_, soil CH_4_ flux; NEE, net ecosystem exchange

^b^Significance is indicated by **P* < 0.050; ***P* < 0.010; ****P* < 0.001
