# Supplementary material for: Microbial Functional Responses Explain Alpine Soil Carbon Fluxes under Future Climate Scenarios
Source: mBio. 2021 Feb 23;12(1):e00761-20. doi: 10.1128/mBio.00761-20 (PMC8545085; doi:10.1128/mBio.00761-20)
Supplement: FIG S3 [file mbio.00761-20-sf003.docx]

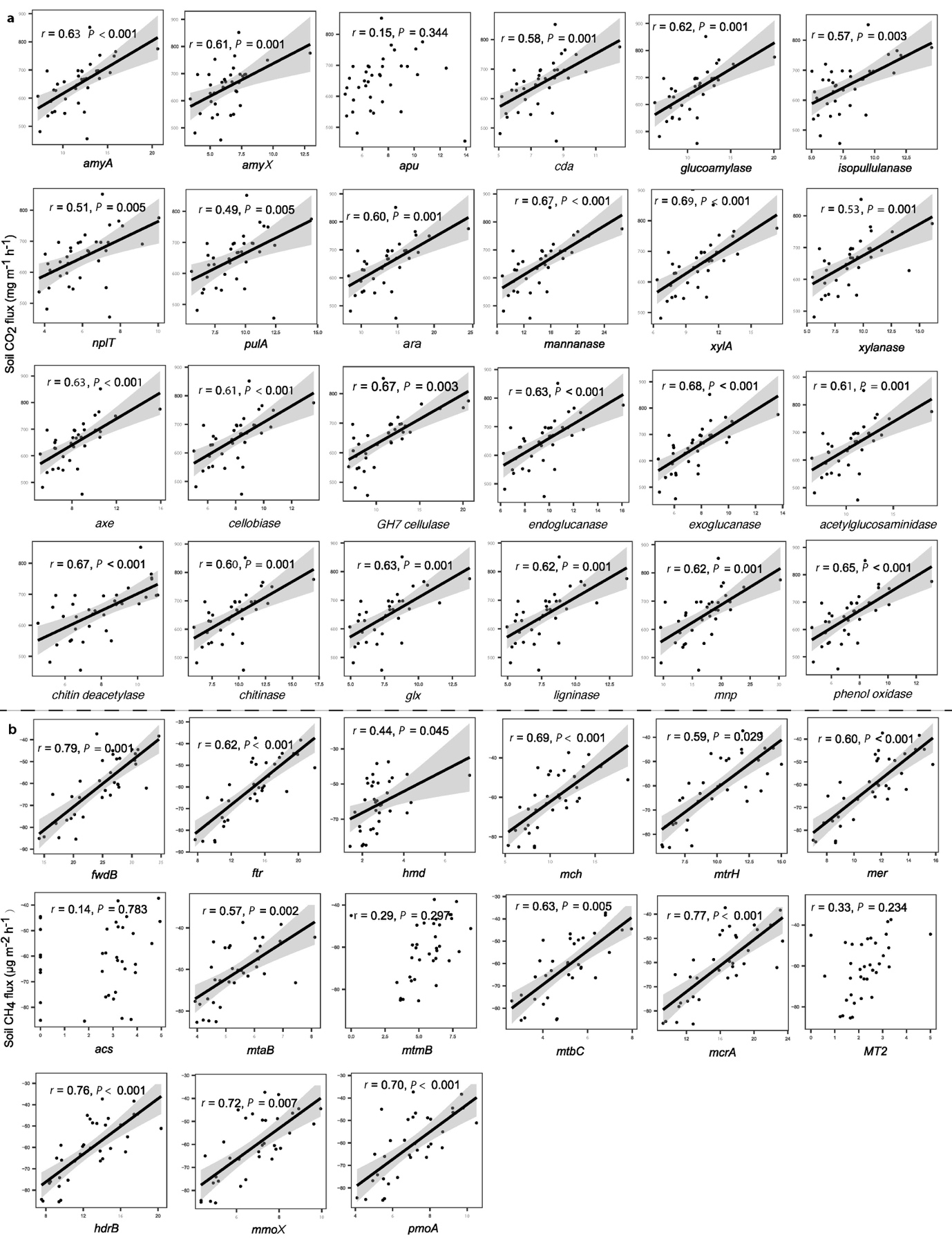


**Fig. S3 Correlations between soil carbon fluxes and the MBC-normalized abundances of microbial functional genes.** Correlations between soil CO_2_ flux and the MBC-normalized abundances of microbial functional genes associated with starch degradation (*amyA*, *amyX*, *apu*, *cda*, *glucoamylase*, *isopullulanase*, *nplT,* and *pulA*), hemicellulose degradation (*ara*, *mannanase*, *xylA,* and *xylanase*), cellulose degradation (*axe*, *cellobiase*, *GH7 cellulase*, *endoglucanase,* and *exoglucanase*), chitin degradation (*acetylglucosaminidase*, *chitin deacetylase,* and *chitinase*), lignin degradation (*glx*, *ligninase*, *mnp,* and *phenol oxidase*) (a)**.** Correlations between soil CH_4_ flux and the MBC-normalized abundances of microbial functional genes associated with methanogenesis (*fwdB*, *ftr*, *hmd*, *mch*, *mtrH*, *mer,* *acs, mtaB*, *mtmB, mtbC, mcrA, MT2,* and *hdrB*) and methanotrophy (*mmoX* and *pmoA*) (b)**.**
