# Supplementary material for: Microbial Functional Responses Explain Alpine Soil Carbon Fluxes under Future Climate Scenarios
Source: mBio. 2021 Feb 23;12(1):e00761-20. doi: 10.1128/mBio.00761-20 (PMC8545085; doi:10.1128/mBio.00761-20)
Supplement: TABLE S5 [file mbio.00761-20-st005.docx]

**Table S5. Linkages between soil carbon fluxes and microbial functional genes, plant variables, and soil variables based on multiple regression on distance matrices**

|  | Functional  composition^a^ |  | Plant | Soil | Model R^2^ | *F* values | Model *P*-value |
| --- | --- | --- | --- | --- | --- | --- | --- |
| CO_2_ | 249.17  (0.038)*^b^ |  | 13.56 (0.002)** | 6.15  (0.149) | 0.23 | 63.55 | 0.001*** |
| CH_4_ | 29.45  (0.019)* |  | 0.83  (0.070) | 2.38  (0.001)*** | 0.19 | 49.87 | 0.001*** |
| NEE | 6.20  (0.048)* |  | 0.13  (0.277) | 0.08  (0.448) | 0.08 | 20.43 | 0.043* |

^a^Microbial functional composition associated with carbon degradation was used to detect linkages with soil CO_2_ flux and net ecosystem exchange (NEE); microbial functional composition associated with methane cycling was used to reveal linkages with soil CH_4_ flux. Soil variables include soil clay content, soil silt content, soil sandy content, soil pH, soil water content, soil total carbon, soil total nitrogen, soil total phosphorus, soil organic carbon, soil nitrate, soil ammonia, soil available phosphorus, dissolved organic carbon and dissolved organic nitrogen. Plant variables include aboveground and belowground net primary production and plant Shannon diversity.

^b^Regression coefficients are shown. The values in brackets are *P*-values from permutation tests. Significance is indicated by **P* < 0.050; ***P* < 0.010; ****P* < 0.001.
