# Supplementary material for: Microbial Functional Responses Explain Alpine Soil Carbon Fluxes under Future Climate Scenarios
Source: mBio. 2021 Feb 23;12(1):e00761-20. doi: 10.1128/mBio.00761-20 (PMC8545085; doi:10.1128/mBio.00761-20)
Supplement: TABLE S6 [file mbio.00761-20-st006.docx]

**Table S6. R^2^ values and standardized coefficients of predictors based on linear mixed-effects models**

| CO_2_^a^ | |  | CH_4_ | |  | NEE | |
| --- | --- | --- | --- | --- | --- | --- | --- |
| R^2^.f | 0.53 |  | R^2^.f | 0.60 |  | R^2^.f | 0.62 |
| R^2^.fr | 0.69 |  | R^2^.fr | 0.60 |  | R^2^.fr | 0.73 |
| Carbon degradation | 0.69***^b^ |  | Methanogenesis | 0.74*** |  | Carbon degradation | 0.19*** |
| ANPP | 0.58* |  | Methanotrophy | 0.34 |  | ANPP | 0.10*** |
| SOC | 0.54 |  | SWC | 0.06 |  | BNPP | -0.02 |
|  |  |  | ST | 0.00 |  |  |  |

^a^Abbreviations: CO_2_, soil CO_2_ flux; CH_4_, soil CH_4_ flux; NEE, net ecosystem exchange; R^2^.f, model R^2^ values considering fixed effects for carbon fluxes (see *Materials and Methods* for details); R^2^.fr, model R^2^ values considering both fixed and random effects; carbon degradation, the MBC-normalized abundance of functional genes associated with carbon degradation; methanogenesis: the MBC-normalized abundance of functional genes associated with methanogenesis; methanotrophy: the MBC-normalized abundance of functional genes associated with methanotrophy; ANPP, aboveground net primary production; SOC, soil organic carbon; SWC, soil water content; ST, soil temperature; BNPP, belowground net primary production. The abundance of functional genes associated with carbon degradation, ANPP and SOC were used to predict soil CO_2_ flux; The abundance of functional genes associated with methanogenesis and methanotrophy, SWC and ST were used to predict soil CH_4_ flux; The MBC-normalized abundance of functional genes associated with carbon degradation, ANPP and BNPP were used to predict NEE. All square-root VIFs are less than 2.

^b^Standardized coefficient values are shown. Significance of standardized coefficient was calculated by ANOVA and indicated by **P* < 0.050; ***P* < 0.010; ****P* < 0.001.
