# Supplementary material for: Microbial Functional Responses Explain Alpine Soil Carbon Fluxes under Future Climate Scenarios
Source: mBio. 2021 Feb 23;12(1):e00761-20. doi: 10.1128/mBio.00761-20 (PMC8545085; doi:10.1128/mBio.00761-20)
Supplement: TABLE S7 [file mbio.00761-20-st007.docx]

**Table S7. Procrustes analyses between microbial functional and taxonomic compositions**

|  | Sum of squares^a^ | *r* | *P* |
| --- | --- | --- | --- |
| GeoChip - 16S^b^ | 0.997 | 0.05 | 0.885 |
| GeoChip - ITS | 0.986 | 0.11 | 0.528 |
| 16S - ITS | 0.878 | 0.34 | 0.019 |

^a^ Sum of squares, Procrustes sum of squares (m12 squared); *r*, the correlation coefficient in symmetric Procrustes rotation; *P*, the significance of a Procrustes analysis calculated by permutation tests.

^b^GeoChip - 16S, the Procrustes analysis between microbial functional composition and bacterial community composition; GeoChip - ITS, the Procrustes analysis between microbial functional composition and fungal community composition; 16S - ITS, the Procrustes analysis between bacterial community composition and fungal community composition.
